# Evaluation of efficacy of alemtuzumab in 5 patients with aplastic anemia and/or myelodysplastic neoplasm

**DOI:** 10.1007/s00508-016-1091-9

**Published:** 2016-10-14

**Authors:** Wolfgang Füreder, Sabine Cerny-Reiterer, Wolfgang R. Sperr, Leonhard Müllauer, Eva Jäger, Ilse Schwarzinger, Klaus Geissler, Peter Valent

**Affiliations:** 1 Department of Internal Medicine I, Division of Hematology & Hemostaseology, AKH, Medical University of Vienna, Währinger Gürtel 18–20, 1090 Vienna, Austria; 2 Department of Clinical Pathology, Medical University of Vienna, Vienna, Austria; 3 Department of Laboratory Medicine, Medical University of Vienna, Vienna, Austria; 4 Department of Internal Medicine V, Hospital Hietzing, Vienna, Austria

**Keywords:** Myelodysplastic syndrome, Paroxysmal nocturnal hemoglobinuria (PNH), Progenitor cells

## Abstract

Patients with aplastic anemia or hypoplastic myelodysplastic syndrome (MDS) may respond to immunosuppressive therapy, including the anti-CD52 antibody alemtuzumab. We analyzed treatment responses to alemtuzumab in 5 patients with MDS or aplastic anemia (AA) evolving to MDS. Two patients with hypoplastic MDS (hMDS) showed a partial response (PR) to alemtuzumab. In both responding patients, a concomitant paroxysmal nocturnal hemoglobinuria (PNH) clone was detected before therapy. One responder relapsed after 15 months and underwent successful allogeneic stem cell transplantation. Both patients are still alive and in remission after 40 and 20 months, respectively. The other patients showed no response to alemtuzumab. One patient died from pneumonia 4 months after treatment. In summary, our data confirm that alemtuzumab is an effective treatment option for a subset of patients with MDS, even in the presence of a PNH clone.

## Introduction

Specific therapeutic options for patients suffering from AA and hMDS are limited. One treatment option is immunosuppressive therapy (IST) [1–4]. Although various forms of IST have been suggested, treatment with anti-thymocyte globulin (ATG) combined with cyclosporine A (CSA) is regarded first-line standard in older patients with AA and those who have no suitable transplant donor [3]. However, ATG/CSA treatment requires hospitalization and is often associated with considerable side effects [3]. In addition, there are patients who do not respond to ATG/CSA or relapse after such treatment. In recent years, the monoclonal CD52 antibody alemtuzumab has been introduced as a new type of IST in patients with AA and hypoplastic MDS [5–10]. Alemtuzumab may be administered intravenously or subcutaneously in these patients. However, the protocols for AA and MDS differ substantially from that used to treat lymphoma patients. Whereas in lymphoproliferative diseases, alemtuzumab is often administered in repeated cycles for several months [11], in AA and MDS patients, the antibody is administered over 5–10 consecutive days in one cycle which may be followed by treatment with CSA [5–10]. This one-cycle alemtuzumab regimen is considered to be better tolerated with less frequent infectious complications.

Several prognostic factors predicting the response to ATG/CSA in patients with AA and MDS have been described. In AA, these include cytogenetic aberrations [12], a (small) paroxysmal nocturnal hemoglobinuria (PNH) clone [13], age, and ‘pretherapy’ blood counts [14, 15]. In MDS, the presence of HLA DR15, younger age, shorter duration of transfusion dependence, pancytopenia, low international prognostic score (IPSS), bone marrow (BM) hypocellularity and—like in AA—the presence of a PNH clone, have been associated with a better outcome [4, 16–18]. No such prognostic factors have been established for AA or MDS patients receiving alemtuzumab as IST so far.

We analyzed ‘pretherapy’ parameters and responses in five patients with dysplastic/aplastic BM who received alemtuzumab as IST.

## Patients and methods

### Diagnostic evaluations

Data of five patients treated with alemtuzumab were analyzed retrospectively. Diagnoses (MDS or/and AA) were established by examining peripheral blood counts, BM biopsy sections, smears, and karyotyping before treatment with alemtuzumab. The numbers of colony-forming progenitor cells (CFU) were studied in all patients. (Table 1). Moreover, patients were examined for the presence of a PNH clone by flow cytometry. The study was approved by the ethics committee of the Medical University of Vienna.

**Table 1:** Blood and progenitor cell counts before therapy

	WBC G/l	ANC G/l	PLT G/l	Hb g/dl	Reti G/l	CFU-GM/ml	BFU-E/ml	CFU-GEMM/ml
#1	2.59	1.2	33	11.8	219.1	24	106	6
#2	1.20	0.54	17	10.6	37.7	0	0	0
#3	1.43	0.60	8	7.5	15.1	12	10	0
#4	1.78	0.71	95	9.0	7.1	1849	42	nk
#5	1.33	0.44	11	13.2	36.4	102	37	3

*WBC* white blood count; *ANC* absolute neutrophil count; *PLT* platelet count; *Hb* hemoglobin level; *Reti* reticulocyte count; *CFU-GM* colony-forming unit granulocyte/macrophage; *BFU-E* burst-forming unit erythroid, *CFU-GEMM* colony-forming unit granulocyte/erythrocyte/monocyte/megakaryocyte; *nk* not known

### Patients’ characteristics and pre-alemtuzumab treatment

Five patients (3 females, 2 males) were included in our study. Their median age at treatment start (alemtuzumab) was 54 years (range 44–69). The time from first diagnosis to alemtuzumab treatment ranged between 1 and 21 years (Table 2). AA and MDS were classified according to published criteria [3, 19, 20].

**Table 2:** Patients’ characteristics

Diagnosis	Gender	Age at diagnosis	Age at start of alemtuzumab	Pretreatment	Transfusions^a^	HLA DR15	Time from diagnosis to alemtuzumab
#1	f	61	64	none	none	nt	3 years
#2	f	46	48	ATG/CSA twice	22	+	2 years
#3	m	68	69	ATG/CSA	42	nt	1 year
#4	f	23	44	ATG/CSA, 5‑Aza	46	+	21 years
#5	m	52	54	ATG/CSA	none	nt	2 years

*nt* not tested; *ATG* antithymocyte globulin; *CSA* cyclosporine A; *5-Aza* 5-azacitidine

^a^transfusions: the number of red cell units transfused during one year prior to start of alemtuzumab are depicted

Four patients had received therapy for AA/MDS prior to alemtuzumab (patients #2, #3, #4, #5). Two of them had failed one cycle of ATG/CSA before being treated with alemtuzumab (#3 and #5), and one patient had received two cycles of ATG/CSA without success (#2). The interval between failed ATG/CSA and alemtuzumab ranged from 6–25 months. Patient #4 was diagnosed with AA in 1990 and treated with ATG/CSA in 1998 which led to a complete remission (CR). After interferon-alpha treatment for hepatitis C, she relapsed in 2008. However, her relapsed AA responded well to re-administration of CSA. In 2011 (21 years after first diagnosis) the patient’s disease evolved to MDS. She was then treated with 5‑azacitidine, albeit without response, and subsequently received alemtuzumab. Patient #1 was treated with alemtuzumab upfront.

### Treatment with alemtuzumab

Four patients (#2, #3, #4, #5) received alemtuzumab according to published protocols for treatment of AA [5]. In particular, alemtuzumab was administered subcutaneously in increasing doses, starting with 3 mg on day 1, followed by 10 mg on day 2, and 30 mg on days 3–5, resulting in a total dose of 103 mg alemtuzumab. Patients #2, #3, and #5 received additional CSA. The dose of CSA was adjusted to achieve a blood trough level of 150–200 µg/l. Treatment was well tolerated, and no dose-reduction or treatment discontinuation was required. Patient #1 was treated with alemtuzumab following a protocol described by Sloand et al. [8] without CSA. After initiation of treatment with 1 mg on day 1, the patient received 3 mg alemtuzumab on day 2 and 10 mg for additional 9 days intravenously, amounting to a total dose of 94 mg alemtuzumab. Premedication consisted of glucocorticosteroids, paracetamol, and histamine receptor blockers.

### Monitoring of laboratory parameters in the follow-up

Follow-up examinations included complete blood counts, serum chemistry, and clinical parameters. In three patients, CFU numbers were recorded in the follow-up, namely in patients #1, #2, and #3. In four patients, BM examination was repeated after alemtuzumab treatment (#1, #2, #3, #5). Chromosome analysis was performed in all patients before therapy; and in three patients (#1, #2, #3), karyotyping was also performed after alemtuzumab therapy. GPI-linked surface markers were analyzed in case of suspected hemolysis (#1, #2, #3, #4).

### Response evaluation

Due to the overlapping nature of the diseases recorded in our patients, responses were classified according to response criteria proposed for AA as well as published response criteria for patients with MDS [21, 22]. Responses according to AA response-criteria were classified as: complete remission (CR), partial remission (PR), and no remission (NR). CR was defined as absolute neutrophil count (ANC) >1.5 G/l, normal hemoglobin (Hb), and platelet counts (PLT) >150 G/l. A PR required transfusion independence and no longer meeting criteria for severe disease. Patients were considered NR when they did not meet criteria for PR or CR. Responses according to MDS criteria were classified as described by Cheson et al. [22]: (1) alteration of the natural history of the disease, (2) cytogenetic response, (3) hematologic improvement.

### Progenitor cell assay

Progenitor cell assays were performed using methylcellulose and colony-stimulating cytokines as described [23]. Based on morphologic appearance and size, colonies were classified as colony-forming unit granulocyte/macrophage (CFU-GM), burst-forming unit erythroid (BFU-E) and colony-forming unit granulocyte/ erythrocyte/ monocyte/ megakaryocyte (CFU-GEMM) .

### Immunohistochemistry

Immunohistochemistry was performed on serial sections prepared from paraffin-embedded, formalin-fixed BM biopsy specimens using the indirect immunoperoxidase staining technique essentially as described [24, 25]. The following antibodies (Ab) were applied: anti-CD34 monoclonal Ab (Clone QBEND10, IgG1 mouse, Novocastra, Newcastle, UK), anti-CXCR4 polyclonal Ab (IgG1 rabbit, Sigma-Aldrich, St. Louis, MO, USA) and anti-VEGF polyclonal Ab (IgG1 rabbit, Santa Cruz Biotechnology, Santa Cruz, CA, USA).

## Results

### Primary response to treatment with alemtuzumab

Five patients with MDS or AA received alemtuzumab; one of these patients was treated with alemtuzumab upfront (#1), whereas four patients received alemtuzumab as second or third line therapy. Two of the five patients achieved a partial remission following alemtuzumab treatment (#2 and #3). Time to PR was 3 and 6 months in patients #2 and #3, respectively according to AA criteria. The other three patients did not respond to alemtuzumab. The patients’ responses to alemtuzumab treatment, according to AA and MDS response criteria, are shown in Table 3.

**Table 3:** Response to alemtuzumab therapy according to AA and MDS criteria

	AA	Natural history	Cytogenetics	HI-N	HI-P	HI-E	IPSS
#1	NR	Failure	NR	na^b^	–	na^b^	1
#2	PR	Stable disease	na^a^	+	+	–^c^	0.5
#3	PR	CR	CCR	+	+	+	1
#4	NR	Failure	nk	+	+	–	1.5
#5	NR	Stable disease	na^a^	–	–	na^b^	0.5

*AA* aplastic anemia; *H-N* hematologic improvement neutrophils; *HI-P* hematologic improvement platelets; *HI-E* hematologic improvement erythrocytes; *IPSS* international prognostic scoring system; *NR* no remission, *PR* partial remission, *CR* complete remission; *CCR* complete cytogenetic response; *PNH* paroxysmal nocturnal hemoglobinuria; *na* not applicable; *nk* not known

^a^The karyotype of patients #2 and #5 was normal throughout the course of the disease

^b^Required pretreatment levels were not met

^c^Patient #2 suffered from hemolysis caused by paroxysmal nocturnal hemoglobinuria

### Posttreatment course and further treatment

As mentioned above, three patients did not respond to alemtuzumab (#1, #4, #5). Patient #4 died from pneumonia 4 months after the start of alemtuzumab. The other two nonresponding patients were further treated with best supportive care. One patient (#2) with a PR developed a relapse 15 months after the start of alemtuzumab. Because of severe thrombocytopenia the patient was treated with eltrombopag, albeit without response. Since the patient had a matched unrelated donor and suffered from life-threatening cytopenia, stem cell transplantation was performed. Apart from a moderate graft-versus-host disease (GvHD), the course was uneventful and the patient remained in CR during the observation period. Patient #3 achieved a continuous PR.

### Survival and progression-free survival

**Fig. 1:**
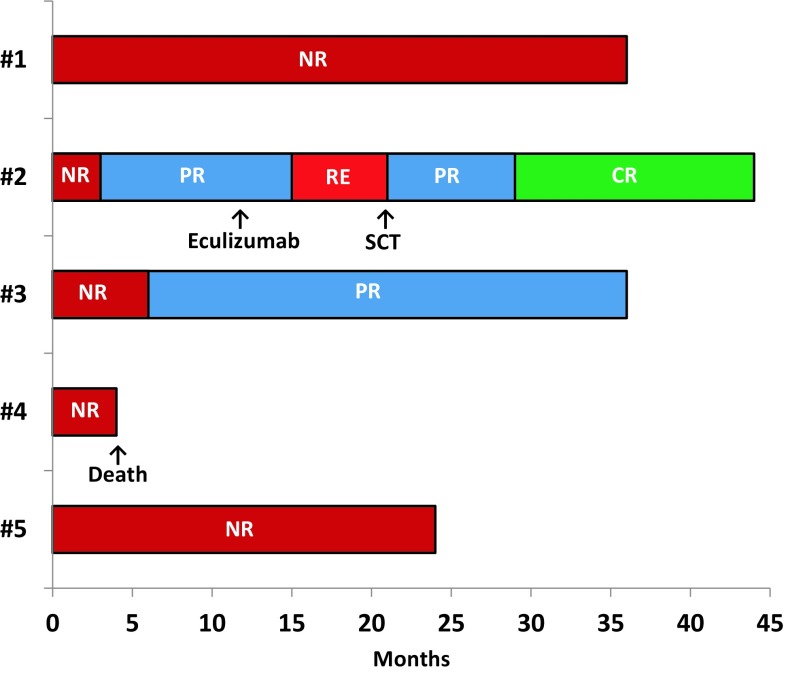
Response and overall survival. Patients #1, #4, and #5 did not respond to alemtuzumab and patient #4 died from pneumonia 4 months after therapy. Patient #2 achieved a PR, relapsed and subsequently underwent allogeneic SCT leading to a CR. In this patient, eculizumab was started 12 months after alemtuzumab and continued until SCT. Patient #3 achieved a continuous PR.* NR* no remission; *PR* partial remission; *RE* relapse; *CR* complete remission; *SCT* stem cell transplantation

All patients had regular follow-up investigations in our departments. Relevant data, including remission status and disease progression, were collected approximately 3 months, 6 months, and subsequently every 6 months after treatment. Patient #3 achieved a PR 6 months after therapy and remains in PR at 36 months. The other responding patient (#2) had a relapse 15 months after alemtuzumab. Subsequently she successfully underwent allogenic SCT and achieved a CR from both AA/MDS and PNH. Three other patients did not respond to alemtuzumab therapy. Patient #4 succumbed to pneumonia even though at the time of her death no granulocytopenia was noted. The MDS of patient #1 progressed to RAEB-1 approximately 3.5 years after alemtuzumab. In patient #5, blood counts remained low but stable and no overt disease progression occurred until he was lost for follow-up 2 years after alemtuzumab. Response to alemtuzumab and survival are shown in Fig. 1.

### Short-term and long-term toxicity of alemtuzumab

Alemtuzumab was well tolerated. Mild drug reactions such as pruritus and rash were noted in three patients (#1, #2, #5). In addition one of them was treated for arterial hypertension (#1). All symptoms were readily kept under control and none of the side effects resulted in dose modifications or treatment interruption. In two patients (#3, #4) no side effects were noted during and shortly after therapy. Patient #2 developed a thyroiditis several months after alemtuzumab treatment. This condition responded well to corticosteroids, but led to a permanent impairment of thyroid function. A relationship between thyre oiditis and alemtuzumab treatment has been described previously [9] and therefore cannot be excluded in our case. Patient #4 died from pneumonia 4 months after alemtuzumab treatment, despite antiviral and antibiotic prophylaxis with valaciclovir and cotrimoxazol. In the other patients, no life-threatening bacterial or viral infections were recorded. No secondary neoplasms were detected in our patients.

### Correlation between clinical/laboratory parameters and treatment responses

No obvious correlations between responses to therapy with alemtuzumab were found when comparing blood counts, karyotypes, HLA DR15 expression, age, gender, transfusion burden, and histologic bone marrow parameters.

### Impact of a concomitant PNH clone

Two patients (#2, #3) developed a PNH clone. In both cases, the PNH clone was detected after treatment with ATG/CSA and before alemtuzumab therapy. In patient #3, the clone size before therapy was 17 % and remained stable at 17 and 21 % at 1 year and 2 years after alemtuzumab start, respectively. A decrease to 6 % in the third year was noted. No substantial hemolysis occurred in this patient. The PNH clone size of patient #2 amounted to 60 % before alemtuzumab therapy and gradually increased to 98 % in the following months. In this patient, a considerable hemolysis was found, so that eculizumab therapy was initiated (12 months after alemtuzumab). This patient was found to respond to eculizumab and was later transplanted.

### Evaluation of colony-forming progenitor cells

Peripheral blood CFU counts were analyzed before and after alemtuzumab in both responding patients (#2, #3) as well as in patient #1. In patient #2, CFU were recorded 2 months prior and 9 months after alemtuzumab. CFU-GM levels increased from 0/ml to 6/ml, BFU-E from 0/ml to 70/ml and CFU-GEMM from 0/ml to 12/ml blood, respectively. Hematologic relapse was accompanied by a decrease in all CFU-subsets to 0/ml blood. In patient #3, CFU-GM levels increased from 12/ml to 15/ml blood, BFU-E from 10/ml to 44/ml, and CFU-GEMM from 0/ml to 5/ml when comparing CFU counts obtained before alemtuzumab treatment with that measured 7 months after therapy. In patient #1, CFU were analyzed 29 months prior to alemtuzumab treatment and 10 months afterwards. CFU-GM increased from 24/ml to 72/ml, while BFU-E (106/ml vs 6/ml) and CFU-GEMM (6/ml vs 0/ml) decreased after treatment in this patient.

### Histology and immunohistochemistry

**Fig. 2:**
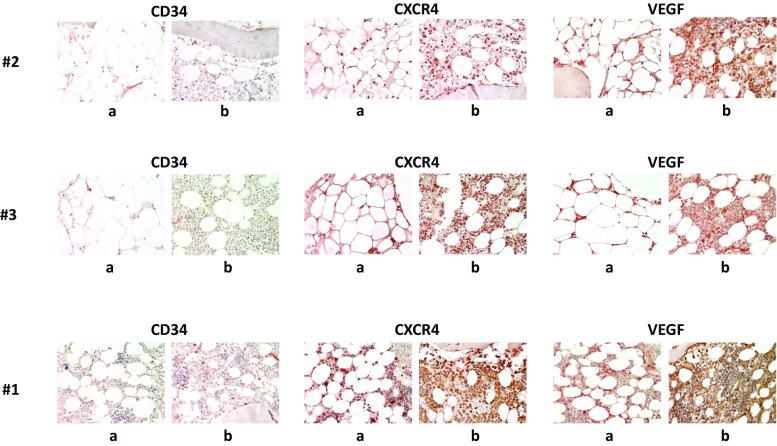
Bone marrow cellularity and microvessel density. Immunostaining using antibodies against CD34, CXCR4, and VEGF of bone marrow sections of three patients before (**a**) and after (**b**) alemtuzumab therapy. Microvessel density and bone marrow cellularity increased following alemtuzumab therapy in both responding patients (#2 and #3) and—surprisingly—also in the nonresponding patient #1

Immunostaining of BM sections, using antibodies against CD34, CXCR4, and VEGF, was performed in three patients before and after alemtuzumab therapy. Pretreatment sections were hypocellular and the BM microvessel density was low (Fig. 2). After therapy, cellularity and microvessel density increased markedly in both responding patients (#2 and #3). Surprisingly however, patient #1 who showed no response to alemtuzumab as defined by AA and MDS criteria, also showed an increase in BM cellularity and microvessel density after treatment (Fig. 2). However, this patient developed RAEB-1 after therapy.

### Cytogenetics

Patients #2 and #5 had a normal karyotype. In patient #1 a trisomy 8 was detected at diagnosis and the karyotype remained unchanged after alemtuzumab treatment. Patient #3 had a normal karyotype at diagnosis, but developed a 13q- with a subclone carrying monosomy 13 after one (unsuccessful) cycle of ATG/CSA. Following alemtuzumab therapy these aberrations disappeared.

Analysis in patient #4 revealed a trisomy 8 and as the disease progressed, an additional isochromosome 17 was detected. No karyotyping was performed after alemtuzmab treatment in this patient.

## Discussion

Alemtuzumab seems to be a promising option for patients who have not responded to or have relapsed after ATG/CSA immunosuppression [5–10]. It is unclear however, whether there are predictive factors for response to this regimen. We describe 5 patients with AA or MDS, who were treated with alemtuzumab. In two of these patients, a remission was obtained, and one patient is still in PR after 36 months, whereas the other patient relapsed and was successfully transplanted using allogeneic stem cells. These data confirm earlier reports and suggest that alemtuzumab remains a treatment option for patients with relapsing AA or/and MDS [5–10].

Different criteria for response to treatment have been described for AA [21] and MDS [22]. However, it seemed problematic to apply the response classification proposed by Cheson et al. for MDS [22] in patients suffering from hMDS. In these patients, blast cell counts are usually low even before therapy and therefore cannot be used to quantify a response. Since the only difference between CR and PR in the MDS response criteria [22] are blast cell counts (‘altering the natural history of the disease’), virtually all patients with improved blood counts that do not reach the levels for a CR also fail to achieve a PR and have to be classified as ‘stable disease’ despite their markedly improved blood counts. Applying response criteria as published for AA [21] might be a useful alternative for response evaluation in hMDS. Alternatively, new response criteria for hMDS need to be established in the future.

A variety of prognostic factors for response to IST with ATG/CSA in AA and MDS have been identified. These include cytogenetic aberrations [12], ‘pretherapy’ blood counts [14, 15], and the presence of a PNH clone [13, 16]. In particular, a small PNH clone has been linked to superior outcome after ATG/CSA treatment in AA as well as in MDS patients [13, 16]. In two of our patients, clonal evolution to overt PNH occurred during ATG/CSA therapy, before alemtuzumab was initiated. Interestingly, these two patients were found to achieve a remission in response to alemtuzumab. Obviously, larger studies with more patients are warranted to clarify whether indeed a PNH clone is indicative of a good response to alemtuzumab second-line treatment in hMDS or AA.

So far, little is known about the value of CFU counting in patients with AA or hMDS in whom IST is applied [26]. However, it is well known that CFU are markedly decreased in these patients. In the present study, we found that pretreatment progenitor cell counts (but not pretherapy blood counts) correlate with the response to alemtuzumab in our patients. In particular, the two patients who responded to alemtuzumab were those with the lowest pretherapy progenitor cell counts, and in both, CFU levels increased during treatment. Again, larger studies are needed to clarify whether progenitor cell counts might be a useful tool to identify patients more likely to profit from alemtuzumab treatment. Thus, it might be possible to exclude patients unlikely to respond to alemtuzumab from this therapy and thereby avoid unnecessary exposure to this IST and the related risk. Infectious complications are a major concern after alemtuzumab therapy. Even though the regimens used to treat AA and MDS are considerable shorter in duration (treatment-days) than those used for CLL, the alemtuzumab-induced immunosuppression may still facilitate infections in AA and MDS patients. No serious infections were noted in four of our patients, but one patient died from pneumonia 4 months after alemtuzumab therapy. It remains unknown however, whether the pneumonia observed in patient #4 was related to treatment-induced immunosuppression. This patient had received standard antiviral and antibiotic prophylaxis after treatment and her neutrophil count had improved substantially after therapy. No pathogen was identified. The dramatic rise of this patient’s neutrophil and platelet counts shortly before her death was unexpected and may or may not have be related to alemtuzumab therapy. Another explanation for the improvement of her blood counts could be a late reaction to prior azacitidine therapy. In addition, the rising counts may also just have reflected an acute phase reaction in connection with the infection that ultimately led to her demise.

Patients in our study were relatively young when compared to typical MDS populations. However, the age of our patients (median 54 years, range 44–69) is similar to that published for other MDS patients treated with IST [2, 4]. One possibility for the younger age in these patients (and our patients) may be the fact that patients with hypoplastic MDS or a MDS-AA overlap type of disease are younger than other patients suffering from classical MDS types. An alternative explanation may be that older patients with such overlapping disease are no longer considered for interventional therapies and are therefore not referred to major hematology centers.

The combination of ATG and CSA is considered standard therapy for AA and hMDS patients who are not eligible for stem cell transplantation [3, 4]. It is unknown whether alemtuzumab in combination with CSA is superior to alemtuzumab alone for treatment of AA or hMDS. Regimens with and without CSA have been shown to be effective in this setting [5–10]. In our study, three patients, including both responding patients, had received CSA concomitantly with alemtuzumab therapy. It seems unlikely that CSA alone was responsible for the remission in our patients since both had received CSA after ATG and relapsed while receiving CSA treatment. However, one might speculate that the combination of CSA and alemtuzumab could be superior to alemtuzumab treatment alone.

In conclusion, alemtuzumab can be administered safely without major side effects and is effective in a subset of patients with aplastic and/or dysplastic bone marrow disorders. Therefore, alemtuzumab therapy should be considered in select patients with hypoplastic MDS. However, larger studies are needed to determine the efficacy and risk of long-term complications of alemtuzumab treatment in AA/MDS patients and to predict what subsets of patients would indeed benefit from this therapy.
